# Pharmacological disruption of the outer limiting membrane leads to increased retinal integration of transplanted photoreceptor precursors

**DOI:** 10.1016/j.exer.2008.01.004

**Published:** 2008-04

**Authors:** E.L. West, R.A. Pearson, M. Tschernutter, J.C. Sowden, R.E. MacLaren, R.R. Ali

**Affiliations:** a Division of Molecular Therapy, University College London, Institute of Ophthalmology, 11-43 Bath Street, London EC1V 9EL, UK; b Developmental Biology Unit, University College London, Institute of Child Health, 30 Guilford Street, London WC1N 1EH, UK; c Molecular Immunology Unit, University College London, Institute of Child Health, 30 Guilford Street, London WC1N 1EH, UK; d Vitreoretinal Service, Moorfields Eye Hospital, 162 City Road, London EC1V 2PD, UK

**Keywords:** retinal transplantation, Müller cell, outer limiting membrane, cell integration, photoreceptor, stem cells, mouse

## Abstract

Retinal degeneration is the leading cause of untreatable blindness in the developed world. Cell transplantation strategies provide a novel therapeutic approach to repair the retina and restore sight. Previously, we have shown that photoreceptor precursor cells can integrate and form functional photoreceptors after transplantation into the subretinal space of the adult mouse. In a clinical setting, however, it is likely that far greater numbers of integrated photoreceptors would be required to restore visual function. We therefore sought to assess whether the outer limiting membrane (OLM), a natural barrier between the subretinal space and the outer nuclear layer (ONL), could be reversibly disrupted and if disruption of this barrier could lead to enhanced numbers of transplanted photoreceptors integrating into the ONL. Transient chemical disruption of the OLM was induced in adult mice using the glial toxin, dl-alpha-aminoadipic acid (AAA). Dissociated early post-natal neural retinal cells were transplanted via subretinal injection at various time-points after AAA administration. At 3 weeks post-injection, the number of integrated, differentiated photoreceptor cells was assessed and compared with those found in the PBS-treated contralateral eye. We demonstrate for the first time that the OLM can be reversibly disrupted in adult mice, using a specific dose of AAA administered by intravitreal injection. In this model, OLM disruption is maximal at 72 h, and recovers by 2 weeks. When combined with cell transplantation, disruption of the OLM leads to a significant increase in the number of photoreceptors integrated within the ONL compared with PBS-treated controls. This effect was only seen in animals in which AAA had been administered 72 h prior to transplantation, i.e. when precursor cells were delivered into the subretinal space at a time coincident with maximal OLM disruption. These findings suggest that the OLM presents a physical barrier to photoreceptor integration following transplantation into the subretinal space in the adult mouse. Reversible disruption of the OLM may provide a strategy for increasing cell integration in future therapeutic applications.

## 1 Introduction

Retinal degeneration is the leading cause of untreatable blindness in the developed world. Current clinical treatments are limited, at best only slowing disease progression and very rarely restoring visual function. Cell transplantation offers a novel therapeutic approach, enabling the replacement of photoreceptor cells lost in the degenerative process. Photoreceptor transplantation may be more feasible than other types of neuronal transplantation, because photoreceptors are stimulated by light and their function is not, therefore, dependent on the reformation of complex afferent connections. Nevertheless, an efferent connection to host second order sensory neurons in the retina is essential for visual function and this is arguably best achieved if the transplanted photoreceptor is fully integrated into the host outer nuclear layer (ONL).

Transplanted whole retinal sheets, derived from either embryonic or neonatal sources, can survive and differentiate, but frequently fail to integrate and make functional connections within the host neural retina (Ghosh and Ehinger, 2000; Royo and Quay, 1959; Seiler et al., 1990; Zhang et al., 2003b). Conversely, dissociated neural stem cells, such as those derived from the adult hippocampus, can migrate extensively within the retina when transplanted into an adult or developing recipient, but rarely differentiate into mature retinal phenotypes (Takahashi et al., 1998; Young et al., 2000). Progenitor cells isolated from dissociated embryonic retinae can differentiate into retinal neurons after transplantation and express photoreceptor-specific markers (Ahmad et al., 2000; Chacko et al., 2000; Coles et al., 2004; Klassen et al., 2004; Qiu et al., 2005; Yang et al., 2002), but migration and integration of these cells into the laminar structure of the host neural retina has remained limited. Better integration has been achieved by transplanting cells into an immature, developing retina, such as that of the neonatal Brazilian opossum, which provides a foetal-like host environment. The ability of transplanted cells to integrate within the host opossum retina declines with host maturation (Sakaguchi et al., 2003, 2004). This also coincides with the maturation of glial elements, such as Müller cells, which form anatomical barriers within the host retina, including the outer limiting membrane (OLM) (MacLaren, 1996).

We have recently shown that a significant degree of integration of fully differentiated and functional photoreceptors can be achieved after transplantation into the adult retina, but only if donor cells are post-mitotic photoreceptor precursors. When transplanted into the subretinal space, these cells can migrate into the recipient ONL, form synaptic connections with downstream targets and are light-sensitive (MacLaren et al., 2006). However, while the number of integrated photoreceptor cells is sufficient to restore the pupillary light reflex, higher levels of integration are needed to improve visual acuity. Given that immature neurons and progenitor cells are intrinsically capable of migrating and differentiating (Komuro and Rakic, 1998a,b; Nadarajah and Parnavelas, 2002; Parnavelas et al., 2002; Pearson et al., 2005), it is likely that natural physical barriers, such as the OLM, impede migration. Transient disruption of these barriers at the time of transplantation might be one way of increasing the number integrating into the host retina.

**Fig. 1:**
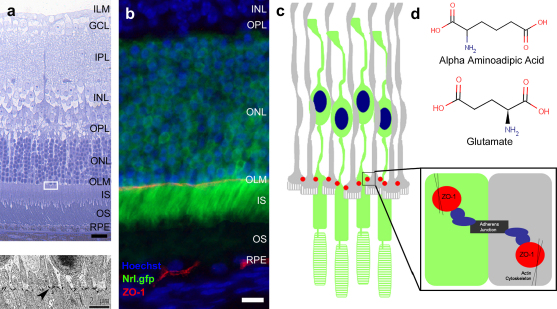
The outer limiting membrane. (a) Semithin section of a wildtype mouse retina, stained with Toluidine Blue showing the location of the outer limiting membrane (OLM). Scale bar, 50 μm. Electron micrograph of the highlighted area shows the electron dense adherens junctions (black arrow head) that form the OLM (insert below). (b) Single confocal image of a retinal section from an Nrl.gfp (green) mouse, stained for zonula occludins-1 (ZO-1; red), an adherens junction protein. Nuclei were counterstained with Hoechst 33342 (blue). Scale bar, 20 μm. (c) Schematic diagram illustrating the adherens junctions (red) that form the OLM, between the cells of the mammalian retina (green photoreceptors; grey Müller cells; blue nuclei). An enlargement of an adherens junction demonstrates the presence of the actin binding protein ZO-1 at the OLM (insert). (d) The chemical structure of alpha-aminoadipic acid (AAA) showing its similarity to glutamate. ILM, inner limiting membrane; GCL, ganglion cell layer; IPL, inner plexiform layer; INL, inner nuclear layer; OPL, outer plexiform layer; ONL, outer nuclear layer; OLM, outer limiting membrane; IS, inner segments; OS, outer segments; RPE, retinal pigment epithelium.

The OLM comprises a series of zonula adherens junctional complexes, located between the plasma membranes of photoreceptor inner segments and the apical processes of Müller glia (Fig. 1a–c). These junctions seal off the light-sensitive photoreceptor inner and outer segments from the rest of the retina, limiting the diffusion of phototransduction cascade components. The OLM is first discernible by post-natal day 5 (P5) in the mouse (Uga and Smelser, 1973; Woodford and Blanks, 1989). Alpha-aminoadipic acid (AAA) is a glutamate analogue (Fig. 1d), which disrupts the OLM by inducing specific toxicity in Müller glial cells within the mammalian retina (Karlsen et al., 1982; Pedersen and Karlsen, 1979). Single intravitreal injections of AAA have been shown to disrupt the OLM irreversibly and to the extent whereby photoreceptors drop out of the ONL to reside amongst outer segments in the subretinal space (Ishikawa and Mine, 1983). In this study, we show that by using an appropriate dose and route of administration, AAA can produce a transient disruption in OLM integrity. Furthermore, OLM disruption can facilitate movement of cells in the opposite direction, significantly enhancing the number of donor photoreceptors integrated into the recipient ONL after transplantation into the subretinal space. These findings suggest that the OLM represents at least one important barrier to cell integration.

## 2 Materials and methods

### 2.1 Animals

C57Bl/6 and Nrl.gfp^+/+^ (Akimoto et al., 2006) mice were maintained in the animal facility at University College London. All experiments were conducted in accordance with the ARVO Statement for the Use of Animals in Ophthalmic and Vision Research. Mice defined as “adult” were at least 6 weeks, but not more than 3 months old. Nrl.gfp^+/+^ mice were used as donors to provide dissociated retinal progenitor cells for transplantation. Recipients were C57Bl/6 animals, unless otherwise stated.

### 2.2 α-Aminoadipic acid formulation and administration

dl-α-Aminoadipic acid (AAA; Sigma) was prepared in phosphate-buffered saline (PBS), adjusted to pH 7.5 and sterile-filtered prior to administration. Mice were anaesthetized with a single intra-peritoneal injection of 0.15 ml of a mixture of Dormitor (1 mg/ml medetomidine hydrochloride; Pfizer Pharmaceuticals, Kent UK), ketamine (100 mg/ml; Fort Dodge Animal Health, Southampton, UK) and sterile water for injections in the ratio of 5:3:42 for adult mice. AAA was administered by intravitreal, subretinal or subcutaneous injection.

For histological assessment, mice were sacrificed at various time points (3–4 mice per time-point) and the eyes were fixed in buffered formalin for 48 h at 4 °C. Retinal sections were prepared by overnight dehydration and paraffin embedding (Histocentre). Sections (5 μm thick) were cut and affixed to glass slides and stained using standard haematoxylin and eosin protocols.

### 2.3 Dissociation of retinal cells and transplantation

To investigate the role of AAA-induced disruption of the OLM on cell integration, C57Bl/6 mice received subretinal transplants of dissociated retinal precursor cells 72 h or 1 week (9 mice per time-point) after intravitreal injection (through the inferior pars plana) of AAA (test eye) or PBS (contralateral eye). Dissociated cells were prepared from P 2–5 Nrl.gfp^+/+^ mice, as described previously (MacLaren et al., 2006). Cells were dissociated using a papain-based kit (Worthington Biochemical, Lorne Laboratories, UK) and diluted to a final concentration of ∼4 × 10^5^ cells/μl. Surgery was performed under direct ophthalmoscopy through an operating microscope, as previously described (MacLaren et al., 2006). Cell suspensions were injected (1 μl) slowly to produce a standard and reproducible retinal detachment in the superior hemisphere. Mice were sacrificed 21 days after transplantation and eyes were fixed in 4% paraformaldehyde (PFA) in PBS, for cell counts. Retinal sections were prepared by cryo-protecting fixed eyes in 20% sucrose, before cryo-embedding in OCT (TissueTek) and sectioning at 18 μm.

### 2.4 Histology and immunohistochemistry

Mice were sacrificed at various time points after AAA administration (3 mice per time-point) and eyes were fixed in 1% PFA in PBS, for immunohistochemistry. Retinal cryosections were permeablized in chilled acetone for 5 min. Sections were pre-blocked in Tris-buffered saline (TBS) containing normal goat serum (5%), bovine serum albumin (1%) and 0.05% Triton X-100 for 2 h before being incubated with primary antibody overnight at 4 °C. After rinsing with TBS, sections were incubated with secondary antibody for 2 h at room temperature (RT), rinsed and counter-stained with Hoechst 33342. Negative controls omitted the primary antibody. The following antibodies were used: rabbit anti-ZO-1 (kind gift of K. Matter) and rabbit anti-GFAP (Dako), with an anti-rabbit Alexa-546 tagged secondary antibody (Molecular Probes, Invitrogen). An apoptosis TdT DNA fragmentation kit (ApopTag Red Apoptosis Detection Kit, Chemicon, CA, USA), which stains apoptotic cells red, was used to perform an *in situ* TUNEL assay on the sections.

### 2.5 Electron microscopy

Mice were sacrificed at various time points after AAA administration. The eyes were fixed, the cornea and lens removed and the eye cups orientated and processed, as previously described (Tschernutter et al., 2005). Ultrathin sections were collected on copper grids, (100 mesh, Agar Scientific) contrast stained with 1% uranyl acetate and lead citrate and analysed using a JEOL 1010 transmission electron microscope (80 kV), fitted with a digital camera for image capture.

### 2.6 Confocal microscopy

Retinal sections were viewed on a confocal microscope (Zeiss LSM510), as previously described (MacLaren et al., 2006). Unless otherwise stated, images show: (i) merged Nomarski and confocal fluorescence projection images of GFP (green) and the nuclear counter stain Hoechst 33342 (blue), and (ii) the same region showing GFP signal only.

### 2.7 Integrated cell counts

Cells were considered to be integrated if the whole cell body was correctly located within the outer nuclear layer and at least one of the following was visible: spherule synapse, inner/outer processes and/or inner/outer segments, as previously defined (MacLaren et al., 2006). The number of integrated cells per eye was determined by counting all the integrated GFP-positive cells in alternate serial sections through each eye. This was doubled to give the mean number of integrated cells per eye. While it is unlikely that a photoreceptor cell body would be present across three sections, given that AAA causes cell swelling in Müller glia (Ishikawa and Mine, 1983), it is possible that it may also affect other cell types including photoreceptors. The total number of nuclei in the ONL in a volume of 2500 μm^3^ at the site of transplantation was determined in both control and AAA-treated eyes (72 h and 1 week prior to transplantation). There was no significant difference in the number of photoreceptor cell bodies between any of the treatment groups (*P* = 0.35, *N* = 6; ANOVA). Thus, AAA treatment is highly unlikely to lead to double-counting of integrated cells.

### 2.8 Apoptotic cell counts

The number of apoptotic cells was determined by counting all TUNEL-positive profiles in each layer of the retina in alternate serial sections. Only sections that encompassed the site of intravitreal injection were used and are thus not representative of apoptosis in the whole eye.

### 2.9 Statistics

All means are stated ±SEM (standard error of the mean), unless otherwise stated. The statistical test used was a two-tailed paired *t*-test with a significance threshold of *P* < 0.05. *N*, number of eyes; *n*, number of sections examined or cells counted, where appropriate.

## 3 Results

### 3.1 Dosage and route of AAA administration

We sought to determine whether it is possible to achieve a transient, reversible disruption of the OLM in the adult mouse by administration of the glial toxin, AAA. First, we assessed different routes of administration including: intravitreal (20 μg/μl, *N* = 4), subretinal (10 μg/μl, *N* = 4) and subcutaneous (0.7–2.7 mg/g body weight, *N* = 6) injection. The dose of AAA used for each route of administration was ascertained from previous published studies (Ishikawa and Mine, 1983; Pedersen and Karlsen, 1979; Rich et al., 1995) Retinae failed to recover normal histological morphology following subretinal injection, while subcutaneous injections resulted in variable morphological changes (data not shown). However, intravitreal administration caused modest and reversible morphological changes (see below). Therefore, AAA was administered to the retina via intravitreal injection, through the inferior pars plana towards the superior hemisphere of the eye, for the remainder of the experiments described.

**Fig. 2:**
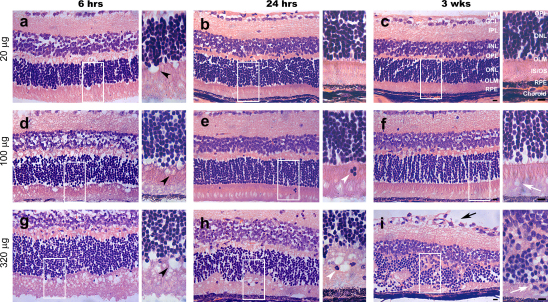
The effect of AAA on adult wildtype retinal morphology. Light images of Haemotoxylin and Eosin (H&E) stained retinal sections from wildtype mice treated with 20 μg, 100 μg and 320 μg of AAA. Mice were sacrificed at 6 h, 24 h and 3 weeks post AAA intravitreal injection. (a, d, g) Vacuoles were present at 6 h post AAA administration in all doses (black arrow heads). (b, c) The retina recovered normal morphology by 24 h, post 20 μg AAA injection. (e, h) Displaced photoreceptor cell bodies suggested outer limiting membrane disruption at 24 h, post 100 μg and 320 μg AAA administration (white arrow heads). (f) Recovery of the retina was observed 3 weeks post 100 μg AAA injection, with a few remaining vacuoles between the outer segments (white arrow). (i) Disruption of the inner limiting membrane (black arrow), loss of retinal layers and degeneration of the photoreceptor inner/outer segments (white arrow), was present 3 weeks post 320 μg AAA injection. Highlighted sections are shown magnified to the right. ILM, inner limiting membrane; GCL, ganglion cell layer; IPL, inner plexiform layer; INL, inner nuclear layer; OPL, outer plexiform layer; ONL, outer nuclear layer; OLM, outer limiting membrane; IS/OS, inner and outer segments; RPE, retinal pigment epithelium. Scale bars, 20 μm.

In order to establish the optimum dose of AAA required to induce a transient disruption of the OLM, several doses (20, 100 and 320 μg/μl, *N* = 3, *N* = 4 and *N* = 4, respectively for each time point) were administered intravitreally and the retinae examined histologically at 6, 24 h or 3 weeks post-injection (Fig. 2) and compared with contralateral controls. At 6 h post-injection of all three doses of AAA, we observed vacuoles at the margin of the OLM that protruded into the inner/outer segment layer. These were possibly due to a swelling of the Müller cell apical processes and both the size and number of vacuoles present was dosage dependent (Fig. 2a,d,g). Upon morphological examination at 24 h post-AAA injection, eyes receiving the low (20 μg/μl) dose appeared normal (Fig. 2b). Conversely, recovery was not evident at this time following application of either 100 or 320 μg doses; vacuoles remained in both, and eyes receiving the highest dose displayed marked morphological abnormalities (Fig. 2e,h). Following administration of these higher doses, the OLM also appeared disrupted, as indicated by the presence of cell bodies in the segment layers (Fig. 2e,h). By 3 weeks post-administration, retinae receiving 20 μg/μl AAA appeared normal by morphological assessment, whilst those receiving 100 μg/μl AAA, exhibited a small number of remaining vacuoles (Fig. 2c,f). In eyes that received the highest dose (320 μg/μl) retinal morphology remained severely disrupted at 3 weeks, including a loss of retinal lamination, sustained disruption of the outer limiting membrane and retinal thinning (Fig. 2i). An apparent disruption of the inner limiting membrane was also noted in these eyes, but was not observed with lower doses of AAA.

Thus, the optimum dose of AAA required for transient OLM disruption by intravitreal injection was determined to be 100 μg/μl. This dose invariably resulted in OLM disruption, as determined by histological assessment, but with consistent recovery of OLM integrity and relatively normal retinal morphology by 3 weeks. The early morphological effects of AAA in the retina described here concur with the few published studies on subcutaneous and intravitreal injections in mice and rats (Pedersen and Karlsen, 1979; Rich et al., 1995). In addition, our findings also demonstrate that these effects can be induced in a reversible manner.

### 3.2 Window of OLM disruption

Having determined the optimal dose of AAA for a reversible and consistent OLM disruption (100 μg/μl, administered intravitreally), we also wished to identify the time point at which this disruption was maximal. We therefore examined retinal morphology, OLM integrity and apoptosis in treated retinae at 24, 48, 72 h, 1 and 2 weeks post AAA administration (*N* = 3 for each time point).

**Fig. 3:**
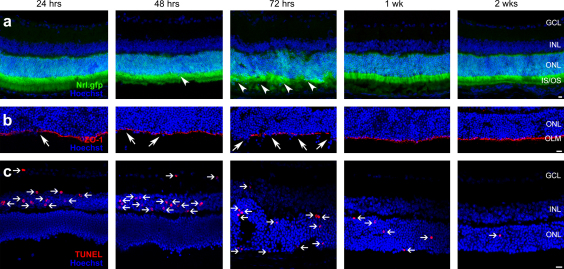
Intravitreal administration of AAA causes transient disruption of the outer limiting membrane. Single confocal images of retinal sections from Nrl-GFP (green) (a) and wildtype (b, c) mice treated with AAA (100 μg) 24, 48, 72 h, 1 and 2 weeks prior to sacrifice. (a) Images show sequential displacement of photoreceptors towards the subretinal space (white arrowheads). The peak of disruption occurred at 72 h and ceased after 1 week post AAA intravitreal injection. (b) Sequential staining for ZO-1 (red), an adherens junction protein, demonstrates some outer limiting membrane disruption at 24 and 48 h, and maximal disruption at 72 h post AAA administration (white arrows). (c) Apoptotic cells, demonstrated by sequential TUNEL staining (red; white horizontal arrows). Nuclei were counterstained with Hoechst 33342 (blue). GCL, ganglion cell layer; INL, inner nuclear layer; ONL, outer nuclear layer; OLM, outer limiting membrane; IS/OS, inner and outer segments. Scale bars, 20 μm.

The effect of AAA pre-treatment on host photoreceptor morphology was examined in the adult Nrl.gfp^+/+^ mouse, which expresses GFP in rod photoreceptors (Akimoto et al., 2006; Mears et al., 2001; Swain et al., 2001). Disruption of the ONL was clearly identifiable by the movement of GFP-labelled cells into the inner and outer segment layers of the retina (Fig. 3a). At 24 h post AAA injection, photoreceptor morphology was largely normal, with correctly orientated inner and outer segments and only occasional photoreceptor cell bodies in the subretinal space. By 48 h, the laminar organization of the retina was disrupted and more photoreceptors were displaced from the ONL. Some retinal folds were evident at 72 h post-injection and the organization of the inner and outer segments was significantly disrupted. Recovery of the retina was first seen at 1 week post AAA injection, as lamination returned and photoreceptor inner and outer segments regained nearly normal orientation. Photoreceptor organization appeared largely normal by 2 weeks post injection.

The displacement of photoreceptor cell bodies into the subretinal space post AAA administration suggested that the OLM was disrupted. To confirm this, we used antibodies directed against ZO-1, an adherens junction protein located at the OLM (see Fig. 1b,c). Staining showed that the OLM was largely intact at 24 and 48 h post-AAA administration, with the exception of a few localized areas of disruption (Fig. 3b). The peak of OLM disruption was present at approximately 72 h post-administration, as demonstrated by a substantial lack of ZO-1 staining at sites where photoreceptors had dropped out of the ONL. By 1 week, the OLM had largely reformed, with disturbed but continuous adherens junctions seen. Normal staining was seen by 2 weeks post AAA administration.

While AAA is a glial-specific toxin (Karlsen et al., 1982; Pedersen and Karlsen, 1979), we wished to establish whether or not other cell types, particularly photoreceptors, might be affected by the changes induced by AAA (Fig. 3c). Retinal sections encompassing the area of AAA administration were stained with an ApopTag Red Apoptosis detection kit and the number of apoptotic nuclei per section quantified. Apoptosis staining revealed that at 24 and 48 h post AAA administration, the vast majority of apoptotic nuclei were present in the inner nuclear layer (25 ± 2 cells per retinal section at 24 h and 31 ± 5 cells per retinal section at 48 h, *n* = 15 sections), most likely Müller cells. Apoptosis was absent in the ONL at these time-points. By 72 h, however, the peak of morphological and OLM disruption, some apoptotic cells were present in the ONL (61 ± 5 cells per retinal section, *n* = 15). Less than 12 ± 2 apoptotic cells per retinal section remained in the ONL at 1 week, reducing to 2 ± 1 cells by 2 weeks post-administration (*n* = 12). Occasionally, apoptotic profiles were seen in the ganglion cell layer. However, these were most likely the result of the intravitreal injection procedure itself, as similar levels were seen in control PBS injected eyes. Note that little apoptosis was observed in any layers of the retina in the rest of the eye, away from the site of AAA injection, at all times points examined. Neuronal toxicity after d,l- AAA treatment is thought to be a secondary effect due to the loss/inhibition of the supporting glial cells in the retina or brain (Tsai et al., 1996). This concurs with our results, since apoptosis in the ONL was observed only after apoptosis of cells in the INL.

**Fig. 4:**
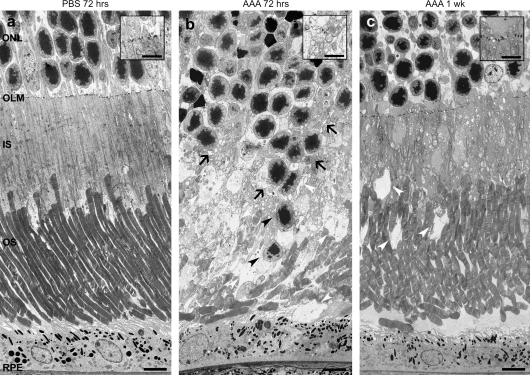
Ultrastructural recovery of the outer limiting membrane 1 week post AAA administration. Electron micrographs of retinal sections from PBS and AAA treated wildtype mice, sacrificed at 72 h (a, b) and 1 week (c) post intravitreal injection. (a) Normal retinal structure was observed in the PBS treated retina and continuous electron dense adherens junctions were present (insert). (b) Disrupted retinal lamination was observed at 72 h post AAA administration. Mislocalized photoreceptors (black arrow heads) and vacuoles (white arrow heads) were present in the segment layer, and a lack of adherens junctions was seen (black arrows and insert). (c) Relatively normal retinal structure was observed 1 week post AAA administration. A few vacuoles remained in the segment layer (white arrow heads) and almost continuous adherens junctions were present (insert). Contrast stained. ONL, outer nuclear layer; OLM, outer limiting membrane; IS, inner segments; OS, outer segments; RPE, retinal pigment epithelium. Scale bars, 5 μm; insert scale bars, 2 μm.

The impact of AAA administration on photoreceptor morphology and OLM integrity were further examined using electron microscopy. Retinae were examined 72 h and 1 week post AAA administration and compared with PBS injected controls (Fig. 4). In PBS injected control retinae, the OLM was intact and photoreceptor inner and outer segment morphology was normal at 72 h post-administration (Fig. 4a). Conversely, the integrity of the OLM was lost in many regions of the AAA treated retinae and photoreceptor nuclei were mislocalized in the outer segment layer. The photoreceptor inner and outer segments were significantly disturbed, vacuoles were present and there was a loss of outer segments (Fig. 4b). By 1 week post AAA treatment, retinae showed significant recovery of OLM integrity, together with fewer vacuoles in the inner segment region and recovery of inner and outer segment organization (Fig. 4c).

Together, these findings demonstrate that intravitreal administration of 100 μg AAA in the mouse causes a transient, reversible disruption of the OLM approximately 72 h post injection.

### 3.3 Cell integration with OLM disruption

We next sought to determine whether or not disruption of the OLM permits greater levels of integration of transplanted photoreceptor precursor cells. To test this, animals received a subretinal injection of early post-natal Nrl.gfp^+/+^ donor cells 72 h after intravitreal administration of AAA, when OLM disruption is maximal. To control for the intravitreal injection, the contralateral eye received a subretinal injection of donor cells 72 h after intravitreal administration of PBS. Three weeks post-transplantation, retinae were sectioned and the total number of Nrl.gfp cells integrated within the ONL was quantified. *Nrl.gfp* expression is restricted to rod photoreceptors (Akimoto et al., 2006; Mears et al., 2001; Swain et al., 2001) and provides genetic evidence that any transplanted integrated cells within the ONL are photoreceptors. We have previously demonstrated that these integrated donor cells are light sensitive and form functional synaptic connections with downstream targets in the recipient retina. Transplanted non-GFP-positive cells remained in the subretinal space, forming a cell mass. Very few integrated cells, that were not photoreceptors, have previously been observed in transplants using donor tissue from C57Bl/6 GFP^+/−^ mice (MacLaren et al., 2006).

**Fig. 5:**
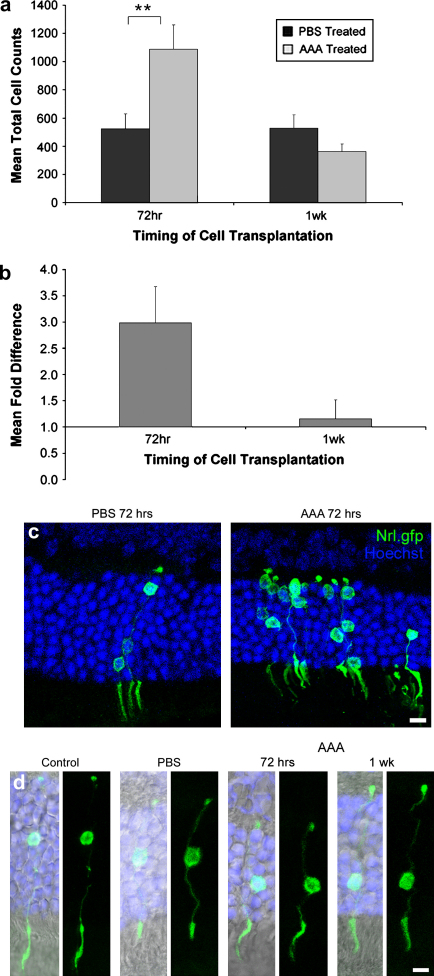
Integration of photoreceptor precursors into retinas with disrupted outer limiting membranes. (a) Number of integrated cells in retinas treated with PBS control or AAA, 72 h and 1 week, prior to cell transplantation (*N* = 9 for each timepoint, **P = 0.009 two-tailed paired *t*-test). (b) Normalized fold-difference in the number of integrated cells for the paired PBS versus AAA treated eyes, 72 h and 1 week prior to cell transplantation. (c,d) Confocal projection images of integrated photoreceptors (Nrl.gfp; green) in wildtype adult retinas, treated with PBS, AAA or a non-treated control, 72 h and 1 week, prior to cell transplantation. Nuclei were counterstained with Hoechst 33342 (blue). Nomarski images are also shown. INL, inner nuclear layer; ONL, outer nuclear layer. Scale bars, 10 μm.

In all animals examined, the eyes that received pretreatment with AAA showed a significantly higher number of integrated donor cells within the ONL compared with their contralateral PBS-treated counterparts (AAA-treated 1088 ± 172.28 cells, vs. PBS control 523 ± 106.49 cells; *N* = 9, *P* = 0.009, paired *t*-test; Fig. 5a). The number of integrated photoreceptors was also increased compared to previous cell count data from wildtype mice (control 691 ± 209.50 cells; *N* = 5). To exclude variability in cell counts resulting from inter-animal variation, the ratio of the number of cells in the AAA treated eye compared with the contralateral control eye was also calculated for each individual mouse. This revealed an average three-fold increase in the number of transplanted photoreceptors for each animal as a result of AAA treatment (3.0 ± 0.75; *N* = 9; Fig. 5b,c).

To determine whether this statistically significant increase in the number of integrated cells coincided with OLM disruption, we also performed cell transplants at 1 week post-AAA administration when the OLM disruption had largely recovered. Transplantation at this stage showed a mean fold-difference of less than 1.3-fold between the AAA treated eye compared with the control PBS-treated eye (Fig. 5b) and the number of integrated cells was not significantly higher post-AAA administration (AAA-treated 363 ± 52.22 cells, vs. PBS control 527.78 ± 94.89; *N* = 9, *P* = 0.34, paired *t*-test; Fig. 5a).

We investigated whether AAA treatment enhanced reactive gliosis as this may affect cell integration. Previous studies have shown that AAA affects astrocytes in both the brain and eye (Ishikawa and Mine, 1983; Khurgel et al., 1996) and Rich et al. (1995) demonstrated up-regulation of GFAP in Müller cell processes, after chronic systemic administration of AAA during development (P3–9). GFAP is a marker of reactive gliosis and astrocytes, and we examined GFAP immunohistochemistry after AAA treatment and compared it with PBS injected controls. Here the doses of AAA used were much lower than those in the studies cited above and were administered as single intravitreal injections. We observed no difference in GFAP staining between the AAA and PBS treated eyes at either time point, indicating that AAA had little effect on Müller cell activation or astrocyte toxicity (Supplementary Fig. 1).

Pre-treatment with either AAA or PBS had no identifiable effect on the morphology of integrated photoreceptors. In both the PBS control and the AAA treated retinae, integrated photoreceptors appeared fully differentiated and morphologically indistinguishable from those we have described previously (MacLaren et al., 2006) (Fig. 5d).

## 4 Discussion

Here we demonstrate that the OLM in the adult mouse retina can be transiently disrupted by the intravitreal administration of AAA. OLM disruption is maximal approximately 72 h post administration. When combined with precursor cell transplantation, this time point correlates with a significantly enhanced level of transplanted photoreceptor cell integration into the recipient ONL, compared with sham-injected controls. These findings suggest that the OLM represents a natural barrier to the successful integration of photoreceptor precursor cells transplanted into the subretinal space. Consideration of the OLM may therefore be important in any future clinical photoreceptor transplantation strategies directed towards retinal repair.

### 4.1 Effect of AAA on Müller cells and the OLM

We show for the first time that the glutamate analogue, AAA, can be used to induce a transient disruption of both Müller glial morphology and OLM integrity in the adult mouse retina. AAA appears to disrupt the OLM by exerting a largely Müller glial-specific transient toxicity (Karlsen et al., 1982; Olney, 1982; Pedersen and Karlsen, 1979). The Müller glia recover well, with only low levels of cell death observed in the first week after AAA administration. Death of Müller glia following exposure to AAA has previously been observed in the carp retina, following an injection of much larger doses than those used in the present study (Sugawara et al., 1990). In addition to Müller cells, the other glial cell type of the retina, astrocytes, also exhibit toxicity in response to AAA in the brain and eye (Ishikawa and Mine, 1983; Khurgel et al., 1996). The mechanism of action of AAA on Müller glia is uncertain, although the morphological damage includes swelling and nuclear changes (Pedersen and Karlsen, 1979). Suggested modes of action include: inhibition of glutamate uptake, resulting in possible neuroexcitotoxicity (Tsai et al., 1996); inhibition of the cystine/glutamate transporter expressed by Müller glia, leading to reduced levels of intracellular glutathione and oxidative stress (Kato et al., 1993); and uptake of AAA itself by Müller glia, and subsequent cytotoxicity via metabolic stress (Chang et al., 1997; McBean, 1994). The downstream effects of AAA occur in a time specific manner, resulting first in the early gliotoxicity, followed by an apparent secondary neurotoxicity, seen with increased dose (Tsai et al., 1996).

### 4.2 OLM disruption and photoreceptor integration

Cells transplanted into the subretinal space of adult mice are capable of correctly integrating within the recipient ONL and forming functional, synaptically-connected photoreceptors, if these cells are at the appropriate post-mitotic precursor stage of development (MacLaren et al., 2006). However, the numbers integrating are below that likely to be required for a clinical therapy. Given that immature neurons and neural stem cells are intrinsically capable of migration (Hagg, 2005; Komuro and Rakic, 1998a; Nadarajah and Parnavelas, 2002; Parnavelas et al., 2002; Pearson et al., 2005), it is likely that barriers exist within the adult retina that impede greater numbers of donor cells from migrating and integrating, as demonstrated by the failure of graft integration in recipients where the host photoreceptor layer is largely intact (Zhang et al., 2003a). Extensive migration into the neural retina is largely restricted either to severely degenerated retina (Zhang et al., 2004) or, in wildtype animals, to areas where there is significant disruption to the ONL (Ghosh et al., 1999; Gouras et al., 1994; Zhang et al., 1999). Zhang and colleagues concluded that breaks in the OLM and/or loss of the photoreceptor component of the OLM were necessary for the formation of bridging fibres between graft and host tissues (Zhang et al., 2003a). Similarly, localized mechanical disruption of the retina may facilitate migration of dissociated cells from the subretinal space, as observed with neural stem cells (Nishida et al., 2000).

The OLM consists of unique heterotypic (involving Müller cells and photoreceptors) or homotypic (between Müller cells) adherens junctions (Paffenholz et al., 1999; Williams et al., 1990). We have shown that AAA causes marked morphological changes within Müller glia, leading to the disruption of these adherens junctions. Furthermore, a number of photoreceptor nuclei became displaced within the segment layer, outside the OLM, which normally acts to retain them (Rich et al., 1995). This suggests that if cells can exit the ONL following disruption of the OLM, the converse is also likely—cells in the subretinal space can migrate more readily into the ONL when the OLM is disrupted. Accordingly, pre-treatment with AAA leads to significantly greater numbers of donor cells integrating following transplantation, compared with controls. Importantly, this effect was only seen if cells were transplanted 72 h after AAA administration, i.e. only when OLM disruption is at its peak.

OLM disruption causes a significant increase in photoreceptor integration following transplantation. However, the increase (3-fold) is less than might be expected if the OLM were the only factor limiting donor cell migration, suggesting that manipulation of additional factors is likely to be required to optimize integration. A number of factors need consideration in relation to our findings. First, the donor cell population is heterogeneous; only a small proportion of cells in the early post-natal retina will be at the appropriate stage and specification for transplantation (i.e. photoreceptor precursors), so cell number may be a limiting factor. This could be augmented in the future by pre-selecting photoreceptor precursors, provided efficient cell sorting methods can be established. Second, Müller glia may actually facilitate donor cell migration into the ONL of the recipient retina or play a role in supporting rod differentiation, and the transient toxic effects of AAA may impede or reduce these supportive functions. Thus, while AAA may aid integration by disrupting the integrity of the OLM, it may conversely limit that enhancement by disturbing this supportive glial scaffold. Müller glia are also known preferentially to support rod process outgrowth (Kljavin and Reh, 1991). However, because all integrated photoreceptor cells in the AAA-treated retinas were morphologically identical to controls and those previously described (MacLaren et al., 2006), this suggests that Müller cells are at least partially dispensable, which is consistent with the observation that AAA-treatment does not affect normal photoreceptor development in early post-natal mice (Rich et al., 1995).

Finally, Müller glia up-regulate the expression of GFAP and other intermediate filament proteins under stress (Bignami and Dahl, 1979; Bjorklund et al., 1985). It is possible that the physiological changes induced by AAA could trigger aspects of the glial scarring pathway, which may subsequently impede the migration of transplanted cells. Recent work investigating retinal cell transplantation by Kinouchi et al. (2003) has demonstrated that cell migration, to the ganglion cell layer is enhanced in mice lacking GFAP and vimentin, two intermediate filament proteins found in reactive Müller glia and astrocytes. Müller cell morphology was stated to appear normal in these mice and spanned the entire retina. They did not, however, observe increased cell integration into other retinal layers, including the outer nuclear layer (Kinouchi et al., 2003).

### 4.3 Therapeutic implications

The results described here demonstrate a proof of concept; namely that disruption of the OLM increases the integration of transplanted photoreceptor precursor cells. However, the compound used, AAA or the l-AAA enantiomer have been shown to have other significant effects in the retina, albeit at higher doses than those used in this study. These include Müller cell necrosis, light insensitivity and suppression of the electroretinographic b-wave (Kato et al., 1990; Pedersen and Karlsen, 1979; Sugawara et al., 1990). Therefore, AAA is highly unlikely to be of therapeutic value. It will be of considerable interest to identify alternative reagents that can induce a specific, reversible disruption of OLM integrity without impacting on the function of the retina. It is important to note that cystoid macular oedema (CME) is a condition seen in the end stages of many diseases of the outer retina, such as retinitis pigmentosa and diabetic maculopathy. Microscopic examination of pathological specimens has shown that CME represents an intra-cytoplasmic swelling (oedema) of Müller cells in the foveal region (Yanoff et al., 1984) which is similar to the effects of AAA described in this study. Based on the results of our experiments, it is therefore not inconceivable that the OLM disruption resulting from CME may make the diseased human fovea a particularly favourable site for future retinal cell transplantation strategies.

## Appendix Supplementary information

Figure 1 Comparison of reactive gliosis in AAA and PBS treated retinas at various time points. Single confocal images of retinal sections from wildtype mice treated with (**a**) PBS or (**b**) AAA (100 mg), 72 hrs, and 1 wk prior to sacrifice. **a, b,** Sequential staining for GFAP (red), marker of reactive gliosis and astrocytes, demonstrates minimal up-regulation of GFAP in Müller cells at both time points. There appeared to be no difference in GFAP staining at both time points between the AAA and PBS treated retinas. GFAP Nuclei were counterstained with Hoechst 33342 (blue). GCL, ganglion cell layer; INL, inner nuclear layer; ONL, outer nuclear layer. Scale bars, 20 μm.
