# Supplementary figures and images for: Pharmacological disruption of the outer limiting membrane leads to increased retinal integration of transplanted photoreceptor precursors
Source: Exp Eye Res. 2008 Apr;86(4):601–11. doi: 10.1016/j.exer.2008.01.004 (PMC2394572; doi:10.1016/j.exer.2008.01.004)

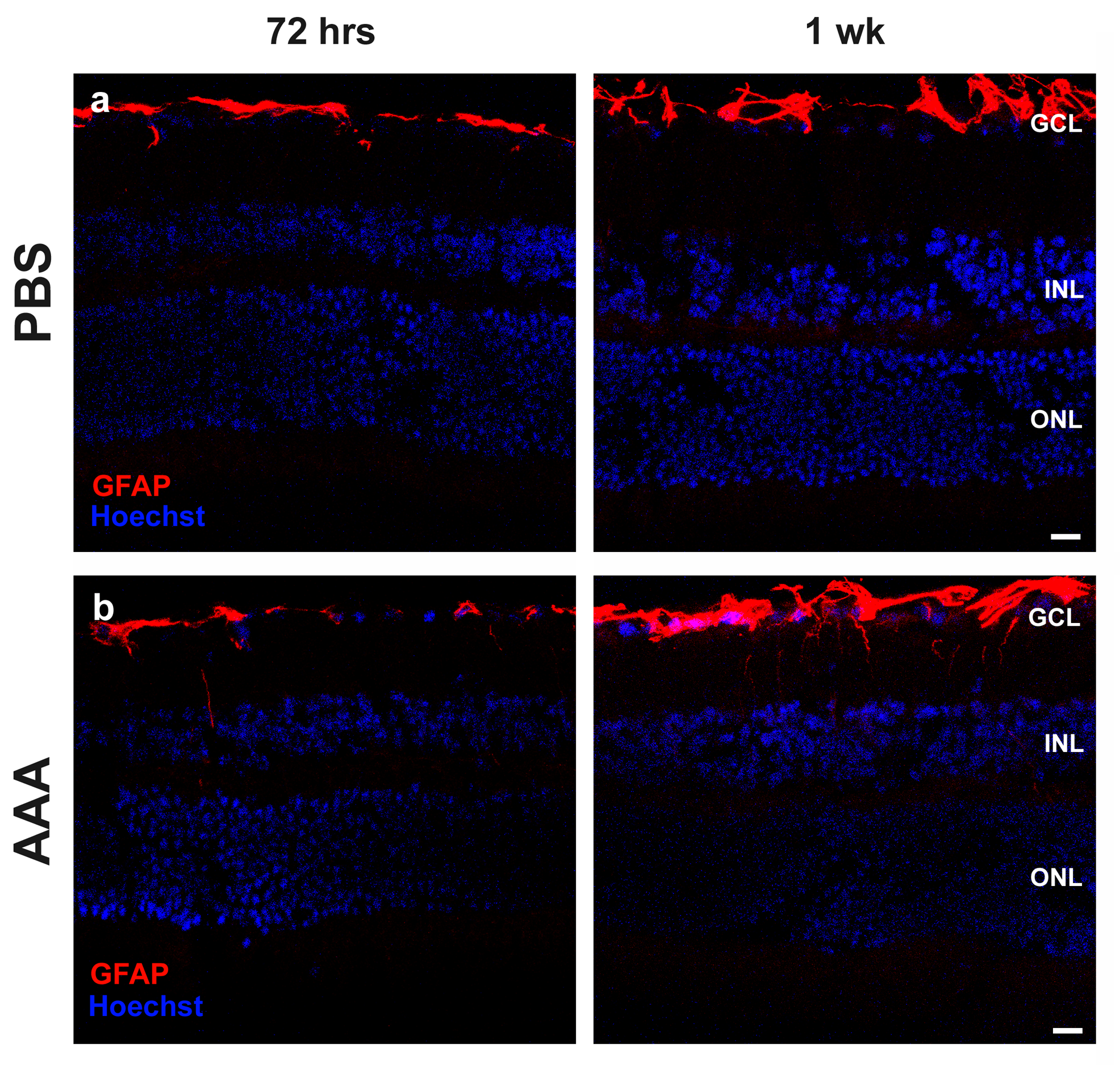

Supplement: Figure 1 — Comparison of reactive gliosis in AAA and PBS treated retinas at various time points. Single confocal images of retinal sections from wildtype mice treated with (a) PBS or (b) AAA (100 mg), 72 hrs, and 1 wk prior to sacrifice. a, b, Sequential staining for GFAP (red), marker of reactive gliosis and astrocytes, demonstrates minimal up-regulation of GFAP in Müller cells at both time points. There appeared to be no difference in GFAP staining at both time points between the AAA and PBS treated retinas. GFAP Nuclei were counterstained with Hoechst 33342 (blue). GCL, ganglion cell layer; INL, inner nuclear layer; ONL, outer nuclear layer. Scale bars, 20 μm. [file mmc1.tif]
